# Characterization of Compounds with Tumor–Cell Proliferation Inhibition Activity from Mushroom (*Phellinus baumii*) Mycelia Produced by Solid-State Fermentation

**DOI:** 10.3390/molecules22050698

**Published:** 2017-04-27

**Authors:** Henan Zhang, Qian Shao, Wenhan Wang, Jingsong Zhang, Zhong Zhang, Yanfang Liu, Yan Yang

**Affiliations:** Institute of Edible Fungi, Shanghai Academy of Agricultural Sciences, National Engineering Research Center of Edible Fungi, Key Laboratory of Edible Fungi Resources and Utilization (South), Ministry of Agriculture, Shanghai 201403, China; henanhaoyun@126.com (H.Z.); shaoqian3697@163.com (Q.S.); wangwenhan@saas.sh.cn (W.W.); 18918162047@189.cn (J.Z.); zhangz0815@126.com (Z.Z.); aliu-1980@163.com (Y.L.)

**Keywords:** *Phellinus baumii*, solid-state, Extracts, compound isolation, proliferation inhibition

## Abstract

The inhibition of tumor-cell proliferationbyan organicsolvent extract from the solid-state fermentation of *Phellinus baumii* mycelia inoculated in rice medium was investigated in vitro. The active compounds inhibiting tumor-cell proliferation were characterized. Results revealed that all (petroleum ether, chloroform, ethyl acetate, and butanol) fractions inhibited tumor-cell proliferation in a dose-dependent fashion. The ethyl acetate extract had the highest inhibitory effecton tumor-cell proliferation, and the butanol fraction had the lowest. Six compounds were isolated and purified from the ethyl acetate extract of *P. baumii* mycelia by the tandem application of silica-gel column chromatography (SGCC), high-speed countercurrent chromatography (HSCCC), and preparative HPLC. These compounds were identified by NMR and electrospray ionization-mass spectrometry (ESI-MS) spectroscopic methods as ergosterol (RF1), ergosta-7,22-dien-3β-yl pentadecanoate (RF3), 3,4-dihydroxy benzaldehyde(RF6), inoscavinA (RF7), baicalein(RF10), and 24-ethylcholesta-5,22-dien-3β-ol (RF13). To further clarify the activity of these compounds, the cell-proliferation-inhibition tests of these compounds on various tumor cells were carried out and evaluatedin vitro. Results suggested that compounds RF6, RF7, and RF10 had potent inhibition effects on the proliferation of a series of tumor cell lines, including K562, L1210, SW620, HepG2, LNCaP, and MCF-7cells. These findings indicated that *P. baumii* mycelia produced by solid-state fermentation in rice canbe used to obtain active compounds with the ability to inhibittumor-cell proliferation.

## 1. Introduction

Cancer is the leading cause of human deaths worldwide [1]. The World Health Organization reports that the number of new cancer patients will increase to 24 million in 2032 [2]. Considering the rising trend of cancer incidence, effective treatment is urgently needed to control tumor-cell proliferation. Currently, chemotherapeutic drugs are still playing an important role in cancer therapy. However, the chemotherapeutic drugs on the market have a strong toxicity and side effects [3]. For example, myopathy and rhabdomyolysis were considered to be the side effects of cerivastatin, forcing Bayer’s withdrawal of the drug from the US market. This event led to questions regarding the safety of statins or all hydroxymethylglutaryl-coenzyme A reductase inhibitors [4]. Therefore, it is urgent to develop natural products as drugs with antitumor potential.

Mushrooms have been utilized as an edible and medical resource in some Asian countries for centuries. Recently, research on the functional components of mushrooms has gradually become a hot topic worldwide. Studies have increasingly demonstrated the potential of mushroom-extracted compounds in the prevention and treatment of cancer [5,6,7]. *P. baumii* is a well-known fungus belonging to the genus *Phellinus* in the Hymenochaetaceae family, and grows on mulberry tree; it has been used traditionally as a folk medicine due to its high biological activities, including anti-tumor, anti-inflammatory, and anti-oxidation characteristics [8,9,10]. The cultivation of fungal mycelia by fermentation has been the major source of fungal materials because of the scarcity of wild *Phellinus* species (spp.). Many studies have shown that the fermentation products of *Phellinus* spp. exhibited powerful biological activities. The extracts from submerged culture products by *P. igniarius* showed a good antioxidant effect [11]. The water-soluble polysaccharide extracted from the cultured mycelium of *P. igniarius* exhibited significant antitumor effect through the enhancement of cell-mediated immunity [12]. The solid-state fermentation of dietary fungus can enhance the production of phenolic antioxidant. Furthermore, new products were formed after fungal fermentation [13]. Our previous study found that the ethanol extracts from solid fermentation of *P. baumii* had good cytotoxicity and antioxidant activity [14]. A comparison of the different mycelium fermentation methods between liquid fermentation and solid-state fermentation revealed that the mycelium from solid-state fermentation inoculated in rice medium contained more flavonoids. This finding indicated that solid-state fermentationcan favor the accumulation of important secondary metabolites [14]. However, the main compounds of the extracts contributing to biological activities are still not clear.

In the present study, *P. baumii* was produced in solid fermentation with medium mainly consisting of rice. Cell proliferation inhibitory effects from the various extracts of *P. baumii* mycelia on various tumor cells were examined in vitro to further identify the compounds with potential antitumor activity. Then, the identification and characterization of active compounds were performed. These compounds might be developed into antitumor medicine by pharmaceutical industries.

## 2. Results and Discussion

### 2.1. The Inhibitory Effects of Extracts on HepG2 Tumor Cells

**Figure 1:**
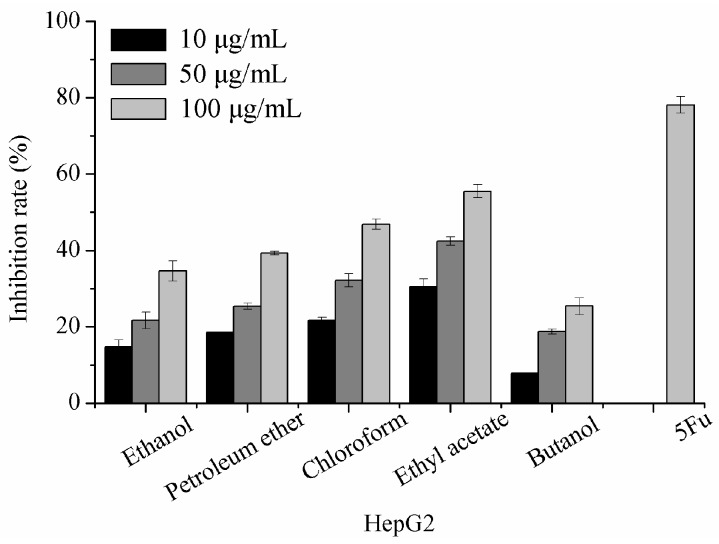
The effect of extracts from *P. baumii* mycelia on the proliferation of HepG2 tumor cells. Each value is expressed as means ± SD (*n* = 3). 5-fluorouracil (5Fu) served as a positive control.

The inhibitory effect of extracts on HepG2 cells proliferation was tested. HepG2 cells were incubated with 10, 50, and 100 μg/mL extracts for 72 h. As shown in Figure 1, the HepG2 cell-growth inhibition of all extracts was in a dose-dependent manner. Butanol extract showed the lowest inhibitory effect, whereas ethyl acetate extract showed the strongest inhibitory effect. Ethyl acetate extract demonstrated an inhibition rate of 55.50% on HepG2 at a concentration of 100 μg/mL, indicating that the compounds composed in ethyl acetate extract exerted potent proliferation that inhibited HepG2 tumor cells.

### 2.2. HPLC Analysis of Extracts from P. baumii Mycelia Fermented on Rice

**Figure 2:**
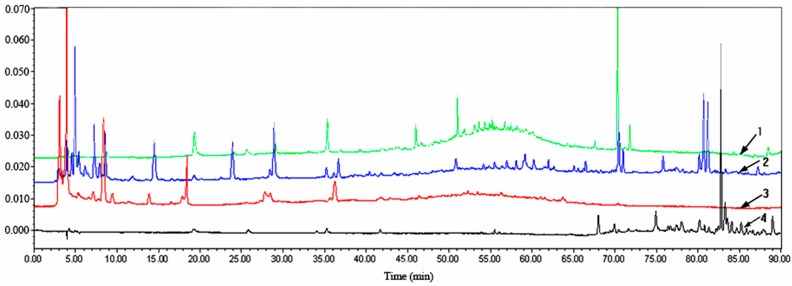
HPLC chromatograms (λ = 254 nm) of the fractions from *P. baumii* mycelium. 1: chloroform extract; 2: ethyl acetate extract; 3: butane extract; 4: petroleum ether extract.

Under the same chromatographic conditions, the HPLC retention maps of different extracts at 254 nm were discrepant, demonstrating that the compositions of these extracts were not the same. As shown in Figure 2, the petroleum ether extract showed a longer peak time, indicating lower polarity, whereas the butanol extract did not. When the retention time was 50 min to 60 min, many complex peaks for the chloroform extract occurred, and the compounds were not separated easily due to their similar polarities. The chromatographic peak time of ethyl acetate extract was shorter, and the peaks appeared in a wide range, demonstrating that the extraction consisted of a rich variety of compounds, having a good degree of separation. As shown in Figure 1, the ethyl acetate extract exhibited the strongest inhibitory effect on tumor cell HepG2, indicating that further isolation and purification of specific cytotoxic compounds is necessary.

### 2.3. Compound Identification and Structure Elucidation

**Figure 3:**
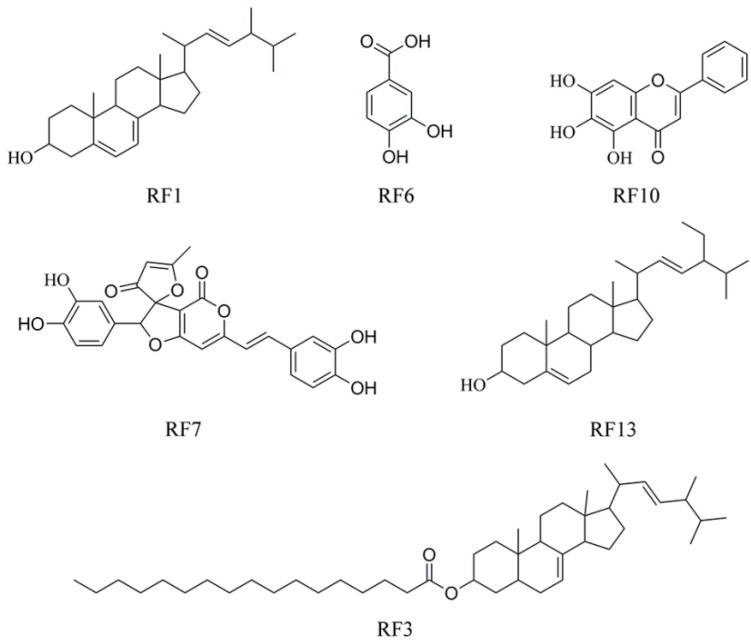
Chemical structures of compounds RF1, RF3, RF6, RF7, RF10, and RF13 isolated from *P. baumii* mycelium produced by solid-state fermentation in rice.

The active fractions from ethyl acetate extract were separated by silica gel column chromatography (SGCC), high-speed countercurrent chromatography (HSCCC), and preparative HPLC to yield active compounds. As shown in Figure 3, the structures of these compounds were resolved by NMR and electrospray ionization (ESI)-mass spectroscopic data with the comparison of previously-reported spectral data [15,16,17,18,19,20,21].

Compound RF1: White crystalline powder with a molecular formula C_28_H_44_O, Mw 396. ^1^H-NMR (500 MHz, CDCl_3_): δ 5.57 (1H, dd, *J* = 4.5 Hz, H-6), 5.38 (1H, t, *J* = 3.5, 7.0 Hz, H-7), 5.23 (1H, dd, *J* = 9.0, 10.0 Hz, H-23), 5.17 (1H, dd, *J* = 10.0, 19.0 Hz, H-22), 3.64 (1H, m, H-3), 2.14 (1H, m, H-20), 0.95 (3H, s, H-19), 0.92 (3H, s, H-25), 0.83 (3H, s, H-27), 0.82 (3H, s, H-28), 0.63 (3H, s, H-18). ^13^C-NMR (125 MHz, CDCl3): δ141.52 (C-8), 139.95 (C-5), 135.73 (C-22), 132.15 (C-23), 119.75 (C-6), 116.45 (C-7), 70.63 (C-3), 55.92 (C-17), 54.73 (C-14), 46.43 (C-9), 42.99 (C-24), 42.99 (C-13), 40.97 (C-20), 40.56 (C-12), 39.26 (C-1), 37.2 (C-10), 38.54 (C-4), 33.26 (C-25), 32.17 (C-2), 28.44 (C-16), 23.16 (C-15), 21.27 (C-21), 21.27 (C-11), 20.1 (C-27), 19.8 (C-26), 17.76 (C-28), 16.45(C-19), 12.21(C-18). Compared with the data published in reference, compound RF1 was identified asergosterol [15].

Compound RF3: White solid with a molecular formula C_43_H_74_O_2_, Mw 622. ^1^H-NMR (500 MHz, CDCl_3_): δ 6.50 (1H, d, *J* = 8.7 Hz, H-6), 6.24 (1H, d, *J* = 8.4 Hz, H-7), 5.24 (1H, dd, *J* = 13.8, 7.8 Hz, H-22), 5.16 (1H, dd, *J* = 13.8, 7.8 Hz, H-23), 3.96 (1H, m, H-3), 1.00 (3H, d, *J* = 6.6 Hz, H-21), 0.91 (3H, d, *J* = 6.9 Hz, H-28), 0.88(3H, s, H-19), 0.84 (3H, d, *J* = 6.6 Hz, H-26), 0.82 (3H, s, H-18), 0.81 (3H, d, *J* = 6.6 Hz, H-27). ^13^C-NMR (125 MHz, CDCl_3_): δ173.7(C=O) 139.7(C-8), 135.9 (C-22), 132.0 (C-23), 117.5 (C-7), 73.3 (C-3), 56.1 (C-17), 55.2 (C-14), 49.4 (C-9), 43.4 (C-13), 43.0 (C-5), 43.0 (C-24), 40.2 (C-20), 39.5 (C-12), 37.0 (C-1), 35.03 (C-4), 34.4 (C-10), 33.2 (C-25), 28.2 (C-2), 27.7 (C-16), 26.9 (C-6), 22.9 (C-15), 21.6 (C-21), 21.3 (C-11), 20.1 (C-26), 19.8 (C-27), 17.8 (C-28), 14.3 (–CH3), 13.1 (C-19), 12.3 (C-18). The spectral data of this compound were consistent with previous reports, and were determined as ergosta-7,22-dien-3β-yl pentadecanoate [16].

Compound RF6: White needle crystal with a molecular formula C_7_H_6_O_3_, Mw 138. ^1^H-NMR (500 MHz, acetone): δ 7.00 (1H, d, *J* = 7.0 Hz, H-5), 7.34 (1H, d, H-6), 7.36 (1H, m, H-2), 8.70 (1H, br s, OH), 8.71 (1H, br s, OH), 9.77 (1H, s, CHO). ^13^C-NMR (125 MHz, acetone): δ 191.1 (1-CHO), 152.3 (C-4), 146.5 (C-3), 131.0 (C-1), 125.5 (C-6), 116.1 (C-2), 115.2 (C-5). Results were basically the same when the published mass spectrometry data of protocatechuic aldehyde were compared, and the compound was identified as 3,4-dihydroxy benzaldehyde [17].

Compound RF7: Yellow amorphous powder, soluble in acetone, methanol, insoluble in chloroform, with a molecular formula C_25_H_18_O_9_, Mw 462. ^1^H-NMR (500 MHz, MeOH-*d*_6_): δ7.32 (1H, d, *J* = 14 Hz, H-7), 7.08 (1H, s, H-9), 7.01 (1H, d, *J* = 8.0 Hz, H-13), 6.79 (1H, d, *J* = 15 Hz, H-6), 6.78 (1H, d, *J* = 8.5 Hz, H-12), 6.73(1H, d, *J* = 8.0 Hz, H-10′), 6.70 (1H, s, H-4), 6.67(1H, d, *J* = 1.5 Hz, H-7′), 6.55 (1H, t, *J* = 1.8 Hz, H-11′), 5.64 (1H, s, H-5′), 5.5 (1H, s, H-2′), 1.98 (3H, s, 1′-CH3). ^13^C-NMR (125 MHz, MeOH-*d*_6_): δ 200.4 (C-3′), 180.1 (C-1′), 175.3 (C-3), 166 (C-5), 158.3 (C-1), 148.5 (C-11), 146.3 (C-9′), 145.6 (C-10), 144.9 (C-8′), 138.4 (C-7), 128.5(C-8), 121.2 (C-13), 121.1 (C-6′), 118.7 (C-11′), 115.9 (C-12), 115.8 (C-6), 115.2 (C-10′), 114.6 (C-7′), 114.4 (C-9), 104.7 (C-2′), 97.1(C-2), 94.8 (C-4), 93.5 (C-5′), 92.1 (C-4′), 16.5 (1′-CH3). Compared with the published data given in references, compound RF7 was identified as inoscavin A [18,19].

Compound RF10: The yellow needle crystal and hydrochloric acid magnesium reaction was cherry red, belong to flavonoids, with a molecular formula C_15_H_10_O_5_, Mw 270. ^1^H-NMR (500 MHz, acetone): δ 12.73 (1H, s, C5-OH), 9.035 (1H, s, C7-OH), 8.0 (2H, m, H-2′, H-6′), 7.6 (3H, m, H-3′, H-4′, H-5′), 6.74 (1H, s, H-3), 6.67 (1H, s, H-8). ^13^C-NMR (125 MHz, CDC_l3_): δ183.4 (C-4), 164.6 (C-2), 153.77 (C-7), 151.52 (C-5), 148.0 (C-9), 132.51 (C-4′), 129.86 (C-6, C-3′, C-5′), 127.13 (C-2′, C-6′), 105.7 (C-3), 105.4 (C-10), 94.7 (C-8). By comparing with previously reported spectral data of the compound, the compound was identified as baicalein [20].

Compound RF13: The compound was obtained by separation, but the purity of the compound was no more than 80%. White crystalline powder with a molecular formula C_29_H_48_O, Mw 412. ^1^H-NMR (500 MHz, CDCl_3_): δ 0.712 (3H, s, H-18), 0.802 (3H, d, *J* = 71 Hz, H-27), 0.825 (3H, t, *J* = 74 Hz, H-29), 0.856 (3H, d, *J* = 66 Hz, H-26), 1.025 (3H, s, H-19). ^13^C-NMR (125 MHz, CDCl_3_): δ 141.3 (C-5), 139.5 (C-22), 129.3 (C-23), 119.5 (C-6), 71.4 (C-3), 55.9 (C-14), 55.7 (C-17), 49.4 (C-24), 48.1 (C-9), 43.5 (C-4), 43.3 (C-3), 42.8 (C-20), 40.8 (C-12), 37.1 (C-1, C-10), 32.0(C-8), 31.4 (C-7), 30.9 (C-25), 29.6 (C-2), 28.3 (C-16), 25.0 (C-28), 21.1 (C-26), 23.0 (C-15), 21.5 (C-21), 20.0 (C-11), 19.4 (C-19), 17.6 (C-27), 13.0 (C-29), 12.1 (C-18). By comparing the published mass spectrometry data of stigmasterol, the compound was identified as 24-ethylcholesta-5,22-dien-3β-ol [21].

### 2.4. Evaluation of the Cytotoxicity of Compounds

In this study, suspension tumor cells (K562 and L1210), hormone-independent solid tumor cells (SW620 and HepG2), and hormone-dependent solid tumor cells (LNCAP and MCF-7) were chosen to evaluate the potential antitumor activity of these compounds.

**Figure 4:**
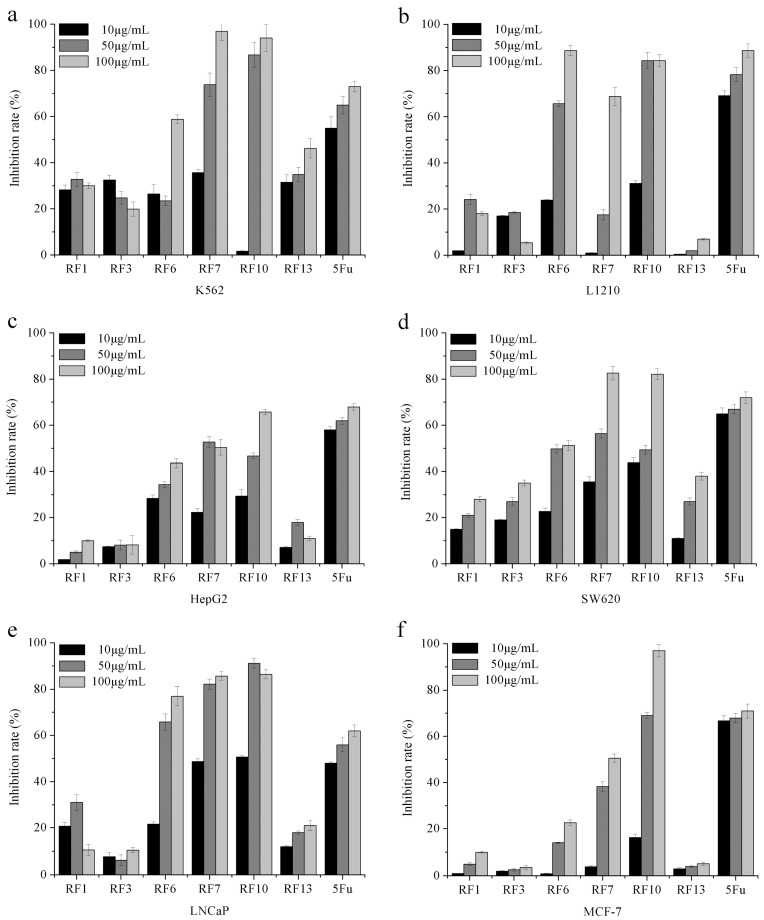
Inhibitory effects of compounds at different concentrations on various tumor cell lines in vitro. (**a**) K562 cells; (**b**) L1210 cells; (**c**) HepG2 cells; (**d**) SW620 cells; (**e**) LNCaP cells; (**f**) MCF-7 cells. 5Fu served as a positive control.RF1 is ergosterol; RF3 is ergosta-7,22-dien-3β-yl pentadecanoate; RF6 is 3,4-dihydroxy benzaldehyde; RF7 is inoscavin A; RF10 is baicalein; RF13 is 24-ethylcholesta-5,22-dien-3β-ol.

K562 is the first human immortalized myelogenous leukemia line. As shown in Figure 4a, RF10 inhibited K562 proliferation in a concentration-dependent manner, and inhibition effects at 50 and 100 μg/mL RF10 were significantly higher than that of positive control 5-fluorouracil (5-Fu). L1210 is another leukemia cell line, derived from the ascitic fluid of 8-month-old female mice. The results showed that the inhibition rates of 50 and 100 μg/mL RF10 were about 84.33% and 84.32%. Similar to the results of leukemia cell lines, the inhibition rates of 100 μg/mL RF10 on HepG2 and SW620 reached about 65.74% and 82.19%, respectively. These values were higher than those of other compounds, and almost equivalent to that of the positive control (5-Fu). LNCaP cells are androgen-sensitive human prostate adenocarcinoma cells, and MCF-7 cells are estrogen-responsive, and often used in vitro to study estrogen receptor-positive breast cancers. As shown in Figure 4e, the inhibitory rates of RF10 at concentration of 50 and 100 μg/mL on LNCaP cells were 91.19% and 86.41%, respectively. These values were significantly higher than that of the positive control group. RF10 showed inhibitory effect on MCF-7 in a concentration-dependent manner, and inhibitory rates of RF10 at concentration of 50 and 100 μg/mL were 69.10% and 97.06%, respectively. RF6 and RF7 were the other two compounds with potent cytotoxicity. As shown in Figure 4e, RF7 had stronger cytotoxicity against K562, SW620, and LNCaP. Furthermore, the inhibition rate of RF7 at a concentration of 100 μg/mL was more than 80%. As shown in Figure 4f, the inhibition rates of RF6 at concentrations of 50 and 100 μg/mL were more than 60% in L1210 and LNCaP. All the results indicated that RF10 had stronger inhibitory activity on tumor-cell proliferation compared with other compounds in this study. The IC_50_ concentrations of RF10 in various tumor cell lines were determined, as shown in Table 1.

Previous findings showed that a variety of phenolic secondary metabolites were isolated from the fruiting bodies of *P. baumii* and exhibited antioxidant activities [22]. Further research indicated that these compounds can inhibit tumor proliferation by interfering with the NF-κB (nuclear factor kappa-B) signaling pathway [23]. Inoscavin A was isolated from mushroom *P. baumii* and exhibited lipoxygenase inhibitory activity [24]. In the previous study, we obtained compound ergosta-7,22-dien-3β-yl pentadecanoate and inoscavin A from *P. baumii* fruiting body, and they were found to inhibit the proliferation of K562 tumor cells [25]. However, wild *P. baumii* fruiting body is rare, and the field cultivation cycle is too long to limit the development and utilization of *P. baumii*. Although liquid fermentation is short, it is not conducive to the accumulation of important secondary metabolites. The phenolic compounds were isolated from solid-state fermentation mycelium of *P. baumii* in rice substrate. Then, their yields were comparable to the extraction from wild *P. baumii* fruiting body. In this study, six compounds were isolated and purified from *P. baumii* mycelium fermented in rice through SGCC, HSCCC, and preparative HPLC. Compared with other three compounds, 3,4-dihydroxy benzaldehyde, inoscavin A, and baicalein showed better inhibition effect on tumor proliferation—especially baicalein, which exhibited strong cytotoxicity against all tumor cell lines examined in this study. Baicalein isolated from *Prunus avium* exhibited cytochrome activity and potent broad-spectrum antifungal activity [26]. Moreover, baicalein from *Ficus racemosa* stem bark possessed antidiabetic, hypolipidemic, and protective effects in albino Wistar rats [20]. The anticancer activity of baicalein was reported in LoVo colon cancer cells, and in their drug-resistant subline LoVo/Dx [27]. The inhibitory activity of these flavones on cell growth and their ability to induce apoptosis and cell cycle arrest were observed [28]. The inhibition of cancer cells by flavonoid derivative observed in this study was consistent with previous reports. However, we did not obtain baicalein from *P. baumii* fruiting body in a previous study. The initial isolation of baicalein in the mycelia of *P. baumii* supports the potential anti-tumor application of *P. baumii*. The observed inhibition of tumor-cell proliferation of the extracted natural 3,4-dihydroxy benzaldehyde (RF6) was consistent with the report indicating that synthesized bi- and tri-benzaldehyde were cytotoxic [29]. Besides, chiral drugs have a great influence on the biological activities and pharmacological properties [30]. The determination of the chiral center in the molecules is important for the study of antitumor activity and subsequent drug development. This work will be carried out in our following study.

The mycelia obtained from solid-state fermentation of *P. baumii* in rice were successfully used to extract those aforementioned compounds for the first time. Our results suggested that the mycelia from solid-state fermentation of *P. baumii* contained multiple polyphenol compounds with cytotoxicity, replacing the fruiting body as a resource of natural active products for application in medicine.

## 3. Materials and Methods

### 3.1. Materials and Chemicals

*P. baumii* strain was provided by the Preservation Center of Fungi, Institute of Edible Fungi, Shanghai Academy of Agricultural Sciences. Six tumor cell lines were applied in this research. K562 (Human chronic myeloid leukemia), SW620 (human colon cancer), HepG2 (human hepatocellular carcinoma), LNCaP (human prostate cancer), and MCF-7 (human breast cancer) were obtained from the Cell Resource Center of Shanghai Institute for Biological Sciences, Chinese Academy of Sciences (Shanghai, China). L1210 (murine leukemia) was obtained from ATCC (American Type Culture Collection, Shanghai, China). RPMI-1640, MEM Eagle, L15 medium, and fetal bovine serum (FBS) were from Gibco (Gibco Co., Grand Island, NY, USA). Dimethyl sulfoxide (DMSO) was purchased from Sigma-Aldrich (St. Louis, MO, USA). 5-fluorouracil (5-Fu), penicillin, and streptomycin were purchased from Amersco Inc. (Solon, OH, USA). All other chemical reagents were analytical reagent grade.

### 3.2. Culture Media and Conditions

The *P. baumii* strain was initially incubated at 26 °C on PDA (Potato Dextrose Agar) slants. After 8 days, five mycelia sections (~5 mm × 5 mm) were excised from the slants and inoculated into solid fermentation medium. Solid fermentation was carried out in culture bottles with rice as the main ingredient for 30 days at 26 °C. Each bottle (200 mL) containing 50 g rice, 1.5 g glucose, 1.8 g KH_2_PO_3_, 0.9 g MgSO_4_·7H_2_O, and 60 mL distilled water was sterilized at 121 °C for 2 h.

### 3.3. The Preparation of the Extracts of P. baumii

Mycelia obtained from the solid fermentation of rice were cleaned, freeze drying, and ground into powder. The mycelium powder was soaked in 10 times the volume of 80% ethanol for 24 h. Ethanol extract was decanted, filtered under vacuum, and then concentrated by a rotary evaporator to obtain the dry extract. The crude extract was dissolved in 20% ethanol solution and extracted successively with the same volume of four different solvents (petroleum ether, chloroform, ethyl acetate and butanol) for 24 h. The extraction was repeated twotimes, and the organic extraction phase was collected.

### 3.4. HPLC Analyses

A sample (10 mg) was dissolved in 1mL methanol and centrifuged (12,000 rpm, 10 min), and then the supernatant was collected by filtration through organic membrane (0.22 μm). The HPLC (Waters 600; Waters, Milford, MA, USA) analysis was performed on a system with a reversed-phase C18 chromatographic column (Pack ODS-AQ; 5 microns, 250 mm × 4.6 mm inner diameter; YMC, Japan) as the chromatographic separation and a Waters 2996 photodiode array detector. The injection volume was 20 μL; the flow rate was 1.0 mL/min; and the column temperature was 30 °C. The mobile phase consisted of 0.01% acetic acid in ultra water (A) and acetonitrile (B); gradient elution (0~5 min, 75% A; 5~30 min, 75% A → 60% A; 30~40 min, 60% A–75% A). The photodiode array detector was set at 254 nm for acquiring chromatograms.

### 3.5. The Isolation of Compounds

**Figure 5:**
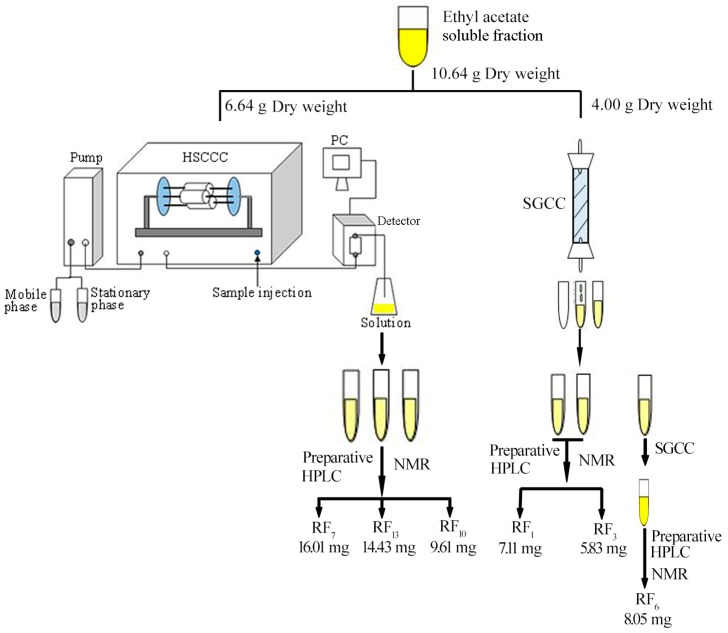
Isolation flow schematic diagram of compounds from ethyl acetate fraction. RF1, ergosterol; RF3, ergosta-7,22-dien-3β-yl pentadecanoate; RF6, 3,4-dihydroxy benzaldehyde; RF7, inoscavin A; RF10, baicalein; RF13, 24-ethylcholesta-5,22-dien-3β-ol. HSCCC: high-speed countercurrent chromatography; SGCC: silica gel column chromatography.

The ethyl acetate soluble fraction was isolated and purified by SGCC (100–200 orders, Qingdao Marine Chemical Inc., Qingdao, China), HSCCC (TBE-300B, Tauto Biotech-nique Company, Shanghai, China), and preparative HPLC to provide compounds. The isolation flow schematic is shown in Figure 5.

Ethyl acetate soluble fraction was separated with SGCC eluted by petroleum–acetone (98:2, 60:40) to obtain sample 1. Sample 2 was obtained from ethyl acetate soluble fraction on SGCC with chloroform–methyl alcohol (95:5, 70:30) as the mobile phase. The fraction of crude 1 was obtained on SGCC eluted by methyl alcohol. Subsequently, crude 1 was separated with SGCC eluted by chloroform–methyl alcohol (98:2, 6:4) as the mobile phase to obtain sample 3. Samples 1–3 were purified by preparative HPLC with MeOH–H_2_O (60:40, 65:35, 42:58) as the mobile phase to obtain compounds A–C, respectively.

In this study, HSCCC was used to separate the cytotoxic compounds from the organic layer of *P. baumii* mycelium, according to our previously reported method with a minor modification [31]. The elution solvent of HSCCC with chloroform, methanol, water, and *n*-butanol (4:3:2:0.5, *v/v/v/v*) was used in this study. The operation condition of HSCCC was selected the upper phase as stationary phase, and the lower phase was used as the mobile phase with a flow of 2.0 mL min^−1^ pump into coiled column. Meanwhile, the apparatus of HSCCC revolution speed and the wavelength of the maximum absorption of UV-500 monitor (Tauto Biotechnique Company, Shanghai, China) were 900 rpm and 254 nm, respectively. Based on the scheme established above, we got samples4, 5, and 6. Samples 4–6 were purified by preparative HPLC with MeOH–H_2_O (46:54, 70:30, 60:30) as the mobile phase to obtain compounds D–F, respectively.

### 3.6. Structure Analyses

The chemical structures of isolated compounds identified by ESI-mass spectrometry were recorded on a Waters Micro-mass GTC spectrometer (Waters Technologies, Milford, MA, USA), NMR. ^1^H-NMR and ^13^C-NMR were recorded on a BrukerAvance 500 NMR spectrometer (Fällanden, Switzerland) operating at 500 MHz (^1^H) and 125 MHz (^13^C), respectively. Chemical shifts were reported in ppm and tetramethylsilane.

### 3.7. Cell Culture and Cell Antiproliferation Assay

#### 3.7.1. Cell Lines and Culture

The tumor cell lines K562 (human chronic myeloid leukemia), HepG2 (human hepatocellular carcinoma), LNCaP (human prostate cancer), SW620 (human colon cancer), and L1210 (murine leukemia) were maintained in RPMI-1640 medium containing 10% fetal bovine serum (FBS) and 100 U/L penicillin and 100 mg/mL streptomycin. MCF-7 (human breast cancer) cells were cultured in MEM Eagle medium containing 10% FBS, 0.01 mg/mL bovine insulin, 100 U/L penicillin, and 100 mg/mL streptomycin, at 37 °C in a 5% CO_2_ humidified incubator.

#### 3.7.2. Inhibitory Effects of Compounds from *P. baumii* on Tumor Cell Proliferation

The harvested tumor cells at 80% confluency were resuspended to a final concentration of 1 × 10^4^ cells/mL (around 10% confluency) in the corresponding medium. Test samples were dissolved in DMSO to the final concentrations of 10, 50, and 100 μg/mL. SW620, HepG2, MCF7, and LNCaP cells (1 × 10^4^ cells/mL, 199 μL) and test sample (1 μL) were added to each well of a 96-well plate. 5-Fu (5 μg/mL) and 5‰ DMSO served as positive and negative controls, respectively. Each treatment had three repeated wells. The medium was replaced with 180 μL no-phenol red medium after the incubation of cells for a further 72 h, and then 20 μL of alamar Blue^®^ was added into each well for another 1–4 h of incubation. Absorbance at 570 nm and 600 nm was measured using a micro ELISA autoreader (Bio-Tek Instruments, Inc., Winooski, VT, USA) when the medium color changed. Because K562 and L1210 cells were suspended, the following operation was needed before the absorbance values were determined: the cells were collected by centrifugation (720 rpm, 6 min), were drawn carefully and discarded, and were added with 180 μL of no-phenol red medium and 20 μL of alamar Blue^®^ into each well of 96-well plate; the 96-well plate was placed to a shaker (80 rev min^−1^) for 10 min. All experiments were performed in triplicate, and the data were presented in the form of mean ± SD. The inhibition rate of samples on tumor cells was calculated according to the following formula:

(1) $$\mathrm{Inhibition}\;\mathrm{rate}(\%)=1-\frac{117216\times A_{570(\mathrm{sample})}-80856\times A_{600(\mathrm{sample})}}{117216\times A_{570(\mathrm{control})}-80856\times A_{600(\mathrm{control})}}\times 100$$

## 4. Conclusions

*P. baumii* is a kind of medicinal mushroom known for its diverse biological activities. This study demonstrated that the organic solvent extracts and isolated compounds from *P. baumii* mycelia produced by solid-state fermentation in rice displayed tumor cell inhibitory activity in vitro. Six compounds (ergosterol, ergosta-7,22-dien-3β-yl pentadecanoate, 3,4-dihydroxy benzaldehyde, inoscavin A, baicalein, and 24-ethylcholesta-5,22-dien-3β-ol) were isolated from the ethyl acetate fraction through SGCC, HSCCC, and preparative HPLC, and their structures were identified. Results indicated that compounds inoscavin A and baicalein had stronger cytotoxicity against all kinds of in vitro tumor cells examined in this study—especially baicalein. However, the detailed mechanism of the role of these compounds needs to be investigated. In general, these results suggested that the mycelia of *P. baumii* obtained by solid-state fermentation in rice may replace the fruiting body as a good candidate for the development of a novel natural anti-tumor-cell proliferation agent in medicine.

## Figures and Tables

**Table 1:** IC_50_ concentration of compound RF10 in tumor cell lines.

			RF10			
IC_50_ (μg/mL)	HepG2	K562	L1210	LNCaP	MCF-7	SW620
	60.22 ± 1.98	39.61 ± 0.36	23.62 ± 0.72	19.515 ± 0.59	37.73 ± 1.23	33.17 ± 1.10
